# Skeletal muscle ATP synthesis and cellular H^+^ handling measured by localized ^31^P-MRS during exercise and recovery

**DOI:** 10.1038/srep32037

**Published:** 2016-08-26

**Authors:** Georg B. Fiedler, Albrecht I. Schmid, Sigrun Goluch, Kiril Schewzow, Elmar Laistler, Fabian Niess, Ewald Unger, Michael Wolzt, Arash Mirzahosseini, Graham J. Kemp, Ewald Moser, Martin Meyerspeer

**Affiliations:** 1 Center for Medical Physics and Biomedical Engineering, Medical University of Vienna, Austria; 2 MR Centre of Excellence, Medical University of Vienna, Austria; 3 Division of Endocrinology and Metabolism, Department of Internal Medicine III, Medical University of Vienna, Austria; 4 Department of Clinical Pharmacology, Medical University of Vienna, Austria; 5 Department of Pharmaceutical Chemistry, Semmelweis University, Budapest, Hungary; 6 Research Group of Drugs of Abuse and Doping Agents, Hungarian Academy of Sciences, Budapest, Hungary; 7 Department of Musculoskeletal Biology and MRC-Arthritis Research UK Centre for Integrated Research into Musculoskeletal Ageing (CIMA), Faculty of Health & Life Sciences, University of Liverpool, UK

## Abstract

^31^P magnetic resonance spectroscopy (MRS) is widely used for non-invasive investigation of muscle metabolism dynamics. This study aims to extend knowledge on parameters derived from these measurements in detail and comprehensiveness: proton (H^+^) efflux, buffer capacity and the contributions of glycolytic (*L*) and oxidative (*Q*) rates to ATP synthesis were calculated from the evolutions of phosphocreatine (PCr) and pH. Data are reported for two muscles in the human calf, for each subject and over a wide range of exercise intensities. 22 subjects performed plantar flexions in a 7T MR-scanner, leading to PCr changes ranging from barely noticeable to almost complete depletion, depending on exercise protocol and muscle studied by localized MRS. Cytosolic buffer capacity was quantified for the first time non-invasively and individually, as was proton efflux evolution in early recovery. Acidification started once PCr depletion reached 60–75%. Initial and end-exercise *L* correlated with end-exercise levels of PCr and approximately linear with pH. *Q* calculated directly from PCr and pH derivatives was plausible, requiring fewer assumptions than the commonly used ADP-model. In conclusion, the evolution of parameters describing cellular energy metabolism was measured over a wide range of exercise intensities, revealing a relatively complete picture of muscle metabolism.

Metabolism in exercising muscle has a highly dynamic evolution, in particular during the first minute of exercise and early recovery. Non-invasive magnetic resonance spectroscopy (MRS) of individual muscles with high temporal resolution is uniquely suited to measure important aspects of this metabolism^1^.

In the first few seconds essentially all of the ATP demand of the exercise is supplied by phosphocreatine (PCr) breakdown, the fastest available source but of limited capacity. The oxidative pathway of ATP synthesis (which we call *Q*) can provide ATP over long periods, but takes in the order of minutes to be fully activated. The glycolytic pathway of ATP synthesis (which we call *L*) is activated on a timescale intermediate between these two, and can provide high ATP flux for a limited period at high exercise intensities, and to a lesser extent at moderate exercise, to bridge the gap between declining PCr breakdown and takeover by *Q*. A schematic overview of the assumed temporal evolution of ATP turnover, PCr and pH is shown in Fig. 1.

A detailed quantitative understanding of metabolic regulation in exercising muscle requires an integrated approach, combining both computational and experimental methods. One limiting factor is the lack of sufficiently detailed, rich data sets. The dynamics of *Q* and especially *L* in early exercise have not yet been defined in detail over a wide variety of exercise conditions. Part of the reason for this is methodological. Freeze-clamp biopsy studies can provide accurate measurements of a range of intracellular metabolites, but their invasive nature severely limits their temporal resolution. Respiratory exchange measurements (VO_2_) are a valuable non-invasive tool, but the data represent a whole-body average, not straightforwardly representative of the exercising muscle^2^. Invasive measurements of VO_2_ (and e.g. lactate production) by arteriovenous difference sampling can be localized to exercising muscle, but are demanding technically and for the subjects. Non-invasive optical methods like near infrared spectroscopy (NIRS) can be used to quantify muscle oxygenation, and some derived quantities such as oxygen consumption rates^3^. Appropriately interpreted in the light of the relevant physiology, MRS is the only non-invasive method that can quantify all three pathways of ATP synthesis, as well as proton efflux (which we call *E*) and intracellular buffer capacity (*β*) *in situ*^4^.

In recent years MRS methodologies advanced technically and have given many new insights into muscle metabolism. In the present study this is extended to the quantification of ATP turnover in individual muscles in individual subjects, by the use of an experimental setup^5^ providing localized acquisition at high signal-to-noise ratio (SNR), and consequently high temporal resolution. This enables the detailed study of these fast changing processes in unprecedented detail. In particular the quantification of *L* benefits greatly, especially during up-regulation in early exercise.

## Results

### Calf muscle metabolism at the same ergometer force (protocol A)

*After exercise at 40% maximum voluntary contraction (MVC) the PCr depletion was typically substantial in gastrocnemius medialis muscle (GM), accompanied by a range of acidification ranging from no pH drop to a pH of 6.4. In contrast, soleus muscle (SOL) showed less PCr depletion in each subject, and consistently without significant acidification (Fig. 2a,c). Calculated maximum oxidative capacity (Q_max_) values were not significantly different between GM and SOL (Fig. 3c).*

When performing plantar-flexion with the same force prescribed on the pedal of the ergometer, the end-exercise PCr depletion was much greater when data acquisition was localized to gastrocnemius medialis (GM) with 77 ± 15%, than in soleus (SOL) with 18 ± 9%. End-exercise pH was 6.78 ± 0.21 in GM vs. 7.04 ± 0.02 in SOL, see Fig. 2a,c. Calculated mitochondrial capacity (*Q*_max_) values were 0.52 ± 0.13 mM/s in GM and 0.50 ± 0.25 mM/s in SOL (cf. Fig. 3). Five subjects with a PCr depletion below 14% were excluded for calculating the mean *Q*_max_ in SOL because such a low dynamic PCr range did not allow for reliable *Q*_max_ quantification. No significant correlation was found between end-exercise PCr depletion and *Q*_max_ of either GM, SOL or data from both muscles pooled (Fig. 3a). A positive correlation was found between end-exercise pH and *Q*_max_ in GM (linear regression *R*^2^ = 0.41, Spearman’s *ρ* = 0.63, *p* = 0.01, cf. Fig. 3b). *Q*_max_ was not significantly different between GM and SOL in a paired t-test (*p* = 0.9, cf. Fig. 3c). The evolution of pH was very similar among all subjects in SOL, rising at the beginning, staying elevated throughout the exercise, and returning to basal levels after the exercise. A very different pH evolution was seen in GM: after a similar initial rise, pH declined throughout the exercise, with a further drop post-exercise. The magnitudes of the initial rise and the post-exercise drop in GM were similar between subjects, while the values of end-exercise pH showed large inter-subject variation, broadly reflecting the respective PCr depletions in each data set. The evolution of pH during and following exercise is shown in Fig. 2c.

### Calf muscle metabolism at the same metabolic state (protocol B)

*When adapting the pedal force in consecutive measurements with the aim of reaching similar end-exercise PCr depletion in GM and SOL, the evolution of both PCr and pH were rather similar between the two muscles (cf. Fig. 2b,d). Q_max_ was found to be higher in SOL than in GM (though at a relatively low number of comparable datasets, cf. Fig. 3d).*

As a consequence of the wide range of exercise intensities in protocol B, the range of end-exercise values obtained was also large: PCr depletion ranged from 16–95% in GM and 4–58% in SOL. End-exercise pH values were between 6.53–7.08 in GM and 7.00–7.09 in SOL. A graph showing the evolution of metabolite concentrations in all subjects would be too complex to be useful, so Fig. 2b,d compare datasets between the two muscles with very similar end-exercise PCr depletion, showing examples of both muscles at low and at medium PCr depletion. Data were selected from four different subjects, to show temporal evolutions with most similar end-exercise PCr depletions. In both regimes PCr and pH evolution were comparable between GM and SOL, and with the measurements from protocol A, at similar values after 3 min of exercise. Three out of the four subjects who reached similar PCr depletion in both muscles showed higher *Q*_max_ in SOL than in GM (cf. Fig. 3d). After pooling data acquired with protocol B and applying a threshold of a minimum PCr depletion of 20%, the average *Q*_max_ was 0.53 ± 0.08 mM/s in GM (12 measurements, from 5 subjects) and 0.96 ± 0.16 mM/s in SOL (9 measurements, from 5 subjects). This difference was statistically significant in an unpaired t-test (*p* < 10^−6^). *Q*_max_ remained significantly higher (*p* < 10^−3^) in SOL (0.80 ± 0.30 mM/s, 14 measurements) than in GM (0.53 ± 0.11 mM/s, 27 measurements) when data from both protocols (PCr depletion >20%) were pooled.

### Relation between pH and PCr

*Acidification started at PCr depletion of 60–75% (cf. Fig. 4c), strikingly independent of exercise intensity, exercise duration or the respective end-exercise values of either PCr or pH, consistently across subjects and muscles.*

Generally, low PCr depletion was associated with neutral to alkaline pH: With protocol A, acidification of GM started during exercise when PCr depletions of 60–75% were reached (cf. Fig. 4a,c). This dependence of starting acidification on momentary PCr depletion was found in all 13 subjects reaching pH < 6.95, except for one subject with falling pH at 50% PCr depletion already. No subject reached acidification in SOL during exercise, with protocol A. In measurements with protocol B both GM and SOL showed similar behavior as seen in GM with protocol A (Fig. 4e,f). The PCr depletion at which pH fell below 7 was independent of end-exercise depletion, exercise intensity and exercise duration, with both measurement protocols (cf. Fig. 4). In early recovery pH declined further, until PCr was replenished to 80%, where pH started to recover towards neutral. The rate in the pH vs. PCr graph increased strongly at 90% of resting PCr (cf. Fig. 4b,d). This dependence of pH changes on PCr level during recovery was independent of time after end of exercise, end-exercise PCr depletion and also absolute end-exercise pH. In measurements with low end-exercise PCr depletion, e.g. in SOL measurements with protocol A, pH recovered towards basal levels on approximately the reverse path it had followed during exercise (cf. Fig. 4b).

### Glycolytic ATP synthesis (*L*)

*In accordance with pH evolution, *L* was only marginally activated in exercises reaching less than about 65% PCr depletion. Above that exercise intensity, L showed substantial increase, correlated with the final end-exercise PCr depletion (cf. Fig. 5a,c). A linear correlation between L and end-exercise values of pH was found (cf. Fig. 5b,d).*

Initial *L* was assessed after 45 s of exercise (grey vertical lines in Fig. 6), representing a practical compromise which allows sufficient time for glycolysis to be activated, but avoids acidification (thus ensuring independence of any assumptions and uncertainties regarding *E*, see Eq. 10). Initial *L* was low in experiments which resulted in minor end-exercise PCr depletion, but increased considerably in those which resulted in at least 60–70% end-exercise PCr depletion (see Fig. 5a), and was correlated linearly with end-exercise pH (Fig. 5b). According to the longer exercise and therefore longer glycolytic activity the measurements from protocol A generally show more acidification at similar initial *L* then their counterparts from protocol B. End-exercise *L* was correlated to PCr depletion and pH in a similar manner (see Fig. 5c,d).

### Temporal evolution of the three pathways of ATP synthesis

*At strong muscle activation L increased rapidly within the first 10 s, had a substantial maximum contribution (up to 50% of overall ATP synthesis, data not shown), and later declined somewhat, but was still about half of its maximum value at the end of exercise (cf. Fig. 6a). The rise of Q was slower, but continued to higher levels, covering the major part of ATP demand at the end of exercise. At lower muscle activation there was little contribution from L, with Q basically mirroring the evolution of PCr splitting (cf. Fig. 6b–d). The alternatively calculated Q_ADP_ (estimated by making the simple assumption of a constant relationship between Q and ADP) shows comparable values, with the notable difference of a faster initial rise at stronger muscle activation.*

The high SNR of the measurements enabled the calculation of individual glycolytic and oxidative ATP synthesis rates. The methods used are most reliable for the beginning and end of exercise, being less dependent on assumptions about the pH-dependence of *E* or the ADP-dependence of *Q*, and less prone to spectrum-to-spectrum fluctuations of pH, to which calculations using the derivative of pH are vulnerable. We therefore present comprehensive data for all measurements in Fig. 5 and examples of full temporal evolution from all regimes of metabolism in Fig. 6.

*L* was calculated as depicted in Eq. 13, using a simple estimation of *E* proportional to ΔpH. Using the assumption of constant ATP demand (*U*), *Q* is calculated from *U*, *L* and *δ*[PCr]/*δt* (see Eq. 14). For comparison the more common approach for calculation of *Q* from the ADP-potential (see Eq. 5) is also included in Fig. 6 (dashed green line).

### Cytosolic buffer capacity

*Individual quantification of cytosolic buffer capacity β using non-invasive ^31^P MRS data was successful, although the calculation is very sensitive to small errors in pH.*

Realistic values were yielded in GM of all subjects from protocol A (group mean ± SD of 16 ± 8 mM/pH unit) and all but one measurement from protocol B (11 ± 5 mM/pH unit). Due to mostly lower muscle activation of SOL the signal change was more difficult to separate from the noise, but still the quantification was successful in 8 subjects of protocol A (group mean ± SD of 24 ± 20 mM/pH unit) and 6 measurements of protocol B (16 ± 17 mM/pH unit). Individual data and averages are shown in Fig. 7. The non- Pi contribution to the buffer capacity *β*_NP_ = 15 mM/pH unit was calculated using the mean *β* in GM, protocol A, to be applied in the subsequent calculations of *L* and *E* of all datasets within this study.

### Proton efflux in early recovery

*At lower acidification the evolution of E showed an increase, whereas at higher acidification E was decreasing. After recovery of acidification to a ΔpH of about −0.2 the E showed a linear correlation with pH (cf. Fig. 8).*

Proton efflux at the beginning of recovery mark temporal changes: muscles with end-exercise acidification (ΔpH > −0.45) showed a fast rise in proton efflux rate *E*, whereas more acidified muscles showed an immediate decline from an initially high *E* (see Fig. 8a). Those muscles that initially increased their *E* well above a rate of 0.05 mM/s experienced a successive decrease of *E*, similar to the initial decrease in the muscles with stronger acidification. Both initial and successive declining of *E* stopped after about 3 min (see Fig. 8c) at values between 0.04 and 0.06 mM/s. Approaching neutral pH the evolution of *E* was approximately linear with ΔpH. This linearity was linked to lowering of the acidification to ΔpH > −0.2, which was reached after 3 min in subjects with low end-exercise acidification (see Fig. 8b) and in later recovery for the higher acidified muscles (see Fig. 8c). Only the two subjects with the highest end-exercise acidification show a decrease of *E* already at higher acidification, although here the evolution towards neutral pH is not known, as at the end of measurement (after 7 min of recovery) pH was still far from recovered. Measurements using protocol B show similar behavior (data not shown). One subjects was excluded from Fig. 8 due to low SNR. Measurements with end-exercise acidification below 0.05 show no efflux (2 subjects).

## Discussion

In this study the evolution of parameters describing tissue metabolism in human calf muscle during exercise and recovery were quantified by localized ^31^P -MRS. Two distinct muscles were studied at a wide range of workloads, densely sampling the range between almost non-existent to (in GM) almost complete PCr depletion. This allowed systematic investigation of the transition from very light to metabolically demanding exercise intensities. From the non-invasively acquired data a detailed analysis of oxidative and glycolytic ATP synthesis as well as proton efflux and cytosolic buffer capacity were performed on a per-subject basis. These calculations were possible only thanks to a high temporal resolution and accurate spatial localization of the acquired MRS data. This, in turn, could only be achieved by using high magnetic field strength (7 Tesla) and an organ specific, optimized RF-coil design to optimize SNR.

### SOL vs. GM

At a given ergometer force soleus showed considerably less PCr depletion than gastrocnemius medialis, and was even working in a different metabolic regime (cf. Fig. 2a,c). This indicates that SOL is activated significantly less than GM in this form of plantar flexion exercise, in accordance with previous studies from our group^5^,^6^,^7^ and others^8^,^9^,^10^. A meaningful comparison of the physiology and metabolite kinetics of the two muscles requires them to be brought to comparable metabolic state as judged by similar end-exercise PCr depletion and acidification. This has not previously been reported in comparable detail and was therefore one of the aims of this study. The results presented in particular in Figs 4 and 5 show consistency between the two muscles over the whole range of exercise intensity (i.e. up to the PCr depletion that was possible to achieve in SOL using the applied protocol).

### Lactate threshold

At low activation of the muscle the ATP need is mostly supplied by PCr breakdown and oxidative ATP synthesis, while at high activation there is a large glycolytic contribution, accompanied by acidification. The transition between these two metabolic regimes corresponds approximately to what is defined in whole body physiology as ‘lactate threshold’ or ‘anaerobic threshold’^11^,^12^,^13^. The relationship between PCr and pH is rather an indirect one. PCr depletion ‘consumes’ protons, but the major contributor to pH alteration during all except low intensity exercise is glycolysis. In our measurements the transition to strong activation of glycolysis appears to happen at PCr depletion of approximately 60–70% (see Fig. 5a,c, cf. Fig. 4c). This was consistent between subjects and muscles, and also across the various exercise intensities: during strong exercise the pH dropped much earlier in time than during medium intensity exercise, but always at the same level of PCr depletion. Beyond around 70% of PCr depletion small differences in muscle activation (or duration of exercise) can result in relatively large differences in activation of *L* and thus acidification (cf. Figs 4 and 5). This can pose difficulties in comparing or averaging measurements.

End-exercise acidification is a consequence of total H^+^ production by *L*, minus proton withdrawal by PCr splitting and *E*. The near-linear relationship of end-exercise pH and initial *L* (Fig. 5b) strongly suggests that *L* is the major defining influence on muscle acidosis, and *E* is either of lower magnitude, has less variance between subjects and exercises, or is regulated coherently with *L*. The correlation of end-exercise pH with end-exercise *L* (Fig. 5d) was to be expected from the mode of calculation (cf. Eq. 13). Both linear correlations are steeper for protocol B, which reflects the need for higher *L* in order to reach the same acidification within a shorter duration of exercise (Fig. 5b,d).

It is not our intention here to discuss the implications of these findings for the regulation of glycolysis in exercising muscle. In general terms, this involves both allosteric-based feedback regulation by metabolites related to energy supply and demand (notably ADP, Pi and AMP), and calcium-related phosphorylation/dephosphorylation cycles involving the key enzyme glycogen phosphorylase which may function in a ‘feedforward’ way^14^. This is still relatively poorly understood in any quantitative way, and (although assumptions of the kind used and discussed here can never be entirely either proved or dispensed with) the application of high spatiotemporal resolution ^31^P MRS to obtain rich datasets *in vivo* is one of the few obvious ways forward.

### Mitochondrial capacity

Calculated *Q*_max_ values are broadly within the range of previous reports^4^,^7^,^15^,^16^. With protocol A, no significant difference of *Q*_max_ was found between the two muscles, although measurements using protocol B showed higher values of *Q*_max_ in SOL. Variation of *Q*_max_ in SOL was higher than in GM of protocol A, and even more so if all measurements are pooled together. This higher variability is perhaps consistent with the literature^17^, reporting GM to be approximately 50% type I fibres. Compared to GM, SOL relies less on glycolytic ATP production, which is broadly consistent with a higher oxidative capacity. In the setup of protocol A SOL was insufficiently active for *Q* to even approach its maximum. When both calf muscles were activated similarly (in protocol B more than 20% PCr depletion was in fact achieved in 9 measurement of 5 subjects, despite a knee angle of nearly 0°), *Q*_max_ of SOL was found to be significantly higher than in GM. However, conclusions about differences in Qmax can only, properly, be tentative, considering the quantitative uncertainties (even after many years of research) in the control relationships between Q and feedback signals like ADP^4^.

*Q*_max_ in GM is approximately stable against end-exercise values of PCr and pH (in SOL there is no data from acidified muscles). The trend of *Q*_max_ vs. pH (cf. Fig. 3) can be interpreted as being a consequence of a muscle with higher *Q*_max_ needing less of *L* to cover the ATP need, and is consistent with studies on trained vs. sedentary subjects^18^,^19^. Much has been made in the ^31^P MRS literature of the relationship of *Q* to [ADP], whose broad characteristics in experiments of this kind is at least consistent with current views of the regulation of oxidative ATP synthesis in skeletal muscle^4^. Figure 3e,f examine this for the first time in detail during the time course of recovery in individual subjects. It may be that the individual variability seen there is evidence that other regulatory mechanisms also contribute^4^.

### Temporal evolution of the three pathways of ATP synthesis

The main result of this study regarding ATP synthesis is the confirmation of a fast initial rise of *L* at stronger muscle activation, with *Q* rising slower than *L*. Further to note is the still considerable contribution of *L* at the end of exercise.

The values of *L*, and particularly more its temporal evolution are not easily accessible. Most methods use an indirect approach, like VO_2_ studies that use whole body oxygen kinetics and power output to make indirect inferences to *L*. Also most ^31^P MRS studies calculate the *L* from overall ATP demand minus contributions from PCr splitting and *Q*_ADP_ (cf. Eqs 5 and 6). On the other hand direct sampling using muscle biopsy is very demanding on the subjects, and limited in temporal resolution and coverage. The applied quantification from the derivatives of pH and PCr (cf. Eq. 13) is technically demanding, as it requires good SNR at high temporal resolution, but the only assumptions needed are the proton stoichiometry of the constituent processes (now well understood), a constant ATP demand (valid in exercise at these sorts of intensity) and the pH-dependence of E (but only in later exercise). In particular, the method is fundamentally free of assumptions regarding regulation of processes or availability of substrates. The dissimilarity of the approach to other methods, together with the direct measurement in the cells of a distinct muscle (vs. measurement further downstream, like blood or exhaled air) makes it an excellent tool for *in vivo* study of particular muscle metabolism, and potentially validation of other methods.

Unlocalized MRS data is often an average over several adjacent muscles, and as a whole body method the VO_2_ data is an average over several skeletal muscles plus cardiac and pulmonary contributions. The specificity of spatially averaged data depends on the distribution of the measured effect in the relevant tissue areas, and also on the question of interrogation. The fast initial rise of *L* and a slower rise of *Q* are in line with general assumptions of muscle metabolism at higher activation, and are also confirmed in biopsy studies^20^. This is also seen in some VO_2_ studies^21^, because the quantified *L* only contains contribution from the muscles where *L* is activated. There is a notable difference in later exercise, where in our measurements at higher muscle activation still a strong contribution of *L* was present, a behavior that was also reported by Conley *et al*.^22^, and might be overshadowed by the averaging over more or less activated muscles in VO_2_ studies^21^. The maximum *L* contribution is much greater in ref. 21, which is an expected consequence of the difference in exercise: 90 s all-out vs. the constant 5 min exercise in our protocol. This might also explain the faster decline of PCr contribution in ref. 21, or again the averaging over muscles in different activation states might dilute the measured effect, which is thus expected to be more accurately quantified in the direct measurement of ^31^P MRS methods.

Quantification of *Q* is expected to be less subject to bias by spatial averaging (as the magnitude and especially the temporal evolution of *Q* is much less dependent on small differences in muscle activation) than *L*, which shows strong change around the lactate-threshold and beyond (cf. Fig. 5). The reported temporal evolution of *Q* throughout exercise and the relation of *Q* to overall ATP demand in later exercise in ref. 21 are similar to our measurements. This at least supports the applicability of our method of calculating *Q* via *L* from temporal derivatives (cf. Eq. 14), as the VO_2_ method is a more direct quantification of *Q*. Other ^31^P MRS studies^19^,^23^ also show similar relations at end of exercise, and as they calculate *Q* equally or similar to *Q*_ADP_ (cf. Eq. 5) they accordingly show the faster initial rise as our alternatively quantified *Q*_ADP_, regarded to be an overestimation of the model in early exercise.

### Cytosolic buffer capacity

To the best of our knowledge this is the first report of non-invasive, individual quantification of cytosolic buffer capacity of human muscle. The accurate calculation of buffer capacity demands sufficient temporal resolution of the transient pH rise before the onset of *L*^24^. Our method, localized ^31^P -MRS at 7 T with a dedicated RF coil, provides raw data with sufficient SNR at a temporal resolution of 6 s. It therefore has the advantage of providing *in vivo* buffer capacity values directly in response to exercise. Literature values of *in vivo* human muscle buffer capacity, to which our data are consistent, and some of the technical difficulties in measuring and comparing them were discussed at length in ref. 14. There have been relatively few measurements reported since then, and this is still a relatively poorly understood aspect^4^. The considerable scattering range of *β* values is expected to be, in part, a consequence of using only the first datapoint for the calculation, a necessity to minimize any contribution of *L*. This is especially so in the less-activated SOL, having smaller initial alkalinization, and thus comparatively bigger influence by noise. The data are included here to demonstrate the feasibility of the method in individuals. If *β* is of major interest, the bias by noise could be reduced by averaging over repeated measurements, which can be performed within a few minutes. Or better, if possible, by using even higher temporal resolution.

### Proton efflux

This is, to our knowledge, also the first study to report the evolution of proton efflux in recovery, quantified in individual measurements at high temporal resolution and, particularly, making early recovery accessible to a measurement (cf. Fig. 8). Assessing *E* is demanding, as it requires good SNR of the fast changing and subsequently declining Pi peak, to accurately quantify pH and its derivative.

At the transition from exercise to recovery many parameters quickly change within the muscle, and the calculations suggest that the effects on the proton efflux from the cell to the blood during early recovery are complicated. *E* is expected to depend on pH primarily, but there are other relevant factors like the gradient of pH between the cytosol and blood: Low perfusion during times of high proton load can lead to acidification of the surrounding blood, which lowers the pH gradient and therefore restricts *E*. Perfusion is known to decrease during recovery, though at higher exercise intensity also a fast initial increase during the first two minutes is reported^25^. A significant residual glycolytic contribution would lead to an underestimation of *E*, as *L* and *E* cannot be separated easily. A residual *L* during recovery would be expected to decline over time, thus the amount by which *E* is underestimated would decline as well, emerging as an apparent increase in *E*. This could potentially explain the initial rise of *E* in the measurements at medium acidification (Fig. 8a). If residual *L* is present during recovery, it is either downregulated differently in higher acidifying muscles, or another effect is acting to decrease *E* more strongly. Comprehensive characterization of *E* in early recovery would greatly benefit from measuring the local muscle perfusion^25^, at a similar time scale. This is possible by MRI^7^,^25^, and the technique to interleave these with ^31^P -MRS in one experiment at 7 Tesla are currently under development^26^.

After the initial recovery phase of about 90 s, both tendencies of rise or fall of *E* abate, suggesting the stabilization of underlying processes. Approaching neutral pH, the validity of the simple model, that *E* is proportional to pH, is supported by the data (pH ≥ 6.8, see Fig. 8). As a consequence this also supports the estimation of *L* evolution during exercise, using an *E* that is modeled by a linear relationship with pH. In early exercise *L* is calculated without any E, and in later exercise sudden changes of metabolic values are not expected. The model of calculating *L* in later exercise relies on calculated initial-recovery *E*, where strong bias can be introduced by averaging. Figure 8a further illustrates the importance of sufficient temporal resolution, which was achieved in our study.

### Strengths and limitations of the work

As a result of the high field strength and the localization scheme, we believe these results offer the clearest and most detailed account so far of the metabolic events relating to ATP turnover and H^+^ handling within the exercising muscle. However, there are significant limitations. A more complicated exercise apparatus and protocol would be required to be sure of minimizing variation in muscle fibre recruitment (due e.g. to small differences in hip rotation). The interactions between contraction and blood flow are complicated and protocol-dependent: we attempted to minimize their effects by acquiring only between the power strokes, but the dynamics of tissue perfusion will still have an influence. Any study of oxidative ATP synthesis would clearly benefit from measurement of muscle cell oxygenation, e.g. by ^1^H MRS of deoxymyoglobin and/or near-infrared spectroscopy (NIRS).

## Conclusion

This study explores subtle details and quick evolutions of metabolic parameters, quantified in individual subjects and muscles, in all metabolic ranges and unprecedented detail. The necessary high SNR was achieved by using an optimized package consisting of a dedicated rf-coil, efficient localization, and a high magnetic field strength of 7 T. In summary, the most important findings were:

- Despite the complexity of the regulation of glycogenolysis in exercising muscle, the **start of acidification was linked to a threshold of PCr depletion**, consistent with the general notion of a ‘lactate threshold’. This was also reflected in quantified levels of glycolysis.
- The **linear correlation of glycolysis to end-exercise pH values** suggests that glycolysis is the main factor influencing muscle acidification.
- **Calculation** of glycolytic and oxidative ATP synthesis rates **from temporal derivatives** of PCr and pH evolution is free of assumptions regarding regulation of processes or availability of substrates. Its successful application in individual non-invasive quantification showed a **faster rise of glycolysis than of the oxidative ATP synthesis** at higher exercise intensities, in line with VO_2_ studies and general assumptions.
- Feasibility of fast individual non-invasive quantification of **cytosolic buffer capacity** was demonstrated, and resulted in reasonable values.
- The swift temporal evolution of **H**^**+**^ **efflux** from the muscle cell cytosol to the blood in early recovery was quantified individually and non-invasively for the first time.

In conclusion, the current study presents a concise quantification of many parameters describing skeletal muscle energy metabolism over a wide range of exercise intensities. A consistent and comprehensive picture of the various stages from the onset of exercise to later stages of recovery is presented, based on localized ^31^P MRS measurements in humans.

## Methods

### Subjects

Twenty-two healthy subjects participated in this study at 26 ± 4 years of age, with BMI of 22 ± 2 kg/m^2^ (both values given as mean ± SD), of which 10 were females. The experimental procedures and protocols were performed in accordance with the Declaration of Helsinki and approved by the Ethics Committee of the Medical University of Vienna. After an explanation of the purpose, nature and potential risks of the study all subjects gave their written, informed consent prior to the experiments.

### Exercise Protocol

Subjects were instructed about the exercise protocol and positioned on the ergometer in the MR scanner. The calf was placed on the form-fitted array coil^27^ and fixed in position using a strap across the tibia. The leg was straight, with the knee extended, similar to a normal standing posture. The setup is described in more detail in ref. 5. Maximum voluntary contraction force (MVC) was measured by repeated isometric pushing against the locked ergometer pedal. The desired force against which plantar flexions were executed (referred to as “ergometer force” in the subsequent text) was set to the respective fraction of the individual’s MVC by adjusting the pressure in the pneumatic system, providing constant force over the pedal’s sweep range. Two different exercise protocols were employed: one with repeated exercise with the same force (40% MVC) to acquire localized data in GM and SOL (protocol A), and one with several different forces (ranging from 20 to 60% MVC), to achieve similar activation in these muscles (protocol B).

#### Protocol A

Fifteen subjects were studied (voxel positions see Fig. 9). Data were acquired for 2 minutes at rest, 5 minutes exercise, and 7 minutes of recovery. During exercise, two consecutive pedal pushes were performed between each data acquisition. With repetition time *T*_R_ = 6 seconds, this resulted in an effective exercise frequency of 0.3 Hz. To minimize habituation effects^28^ and standardize exercise conditions, an equivalent preparatory exercise bout was executed 40 min before acquiring MRS data during exercise. The exercise bouts for acquiring data in GM and in SOL were separated by 28 minutes of rest on average (minimum was 20 min), to ensure that PCr, Pi and pH values were fully recovered to basal levels at the beginning of the second bout. In this form of plantar flexion exercise, with a straight knee and a moderate exercise frequency, predominantly gastrocnemius is expected to be active^29^, and SOL far less. This was also confirmed by perfusion sensitive MRI, applied in a recent study with the same exercise protocol and setup^25^.

#### Protocol B

Eight subjects were measured with protocol B (one subject was taking part in both protocols). The exercise was similar to protocol A, but each measurement comprised several bouts at different levels of exercise, increasing from bout to bout. To keep total measurement time acceptable, the bouts were shorter than in protocol A, after 1 min rest, 3 min exercise and 4 min recovery followed.

### MR spectroscopy

Localized ^31^P -MRS signals were acquired using a semi-LASER sequence^30^,^31^ with a repetition time (*T*_R_) of 6 s. The volumes of interest (VOI) were selected in double oblique orientation and made as large as possible, individually for each muscle in each subject, keeping sensitivity and specificity high (see Fig. 9). Acquired spectra were processed with the jMRUI software package^32^, using the AMARES time domain fit algorithm^33^. PCr and Pi signal intensities were normalized to baseline PCr concentrations, as described elsewhere^5^. Average voxel volumes were 37 ± 5 cm^3^ in GM and 40 ± 10 cm^3^ in SOL, respectively.

### Filtering of the data

Temporal filtering was applied to the evolution of metabolites during and following exercise, in order to improve quantification analysis, e.g. in the computation of derivatives. For pH, where even small but fast fluctuations by slight inaccuracy of Pi peak frequency can induce big fluctuations in the derivative of pH, a moving median filter followed by a moving mean filter was applied. Due to the higher SNR, for PCr data only the moving mean filter was used. Window size for both filters was three data points. Calculated values were checked for bias caused by filtering, and shown to be consistent with values obtained from unfiltered data, but more robust for automated computation.

### Schematic of metabolic time courses

**Figure 1:**
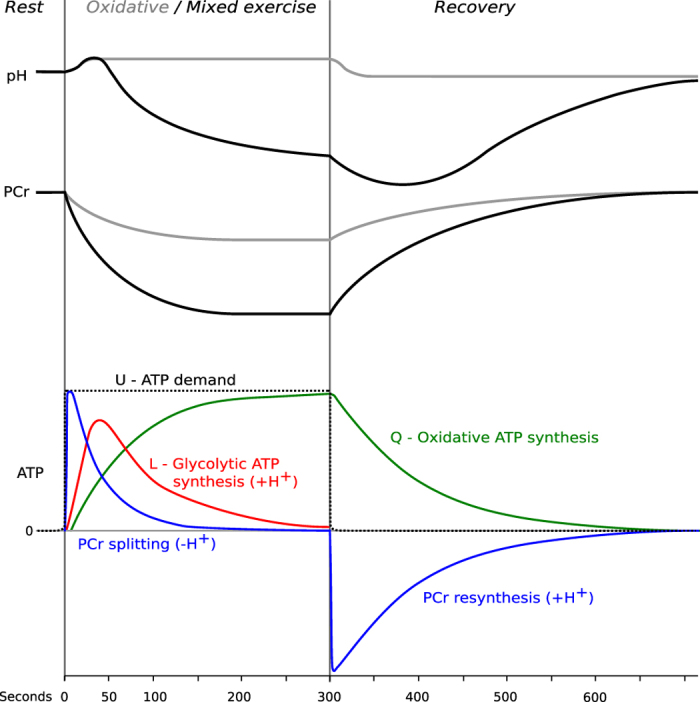
Schematic overview of the common understanding of the temporal evolution of muscle PCr concentration, cytosolic pH and ATP synthesis rates during rest, exercise and recovery (as e.g. in ref. 21). Grey PCr and pH lines depict what one might call ‘pure oxidative’ exercise, i.e. exercise at relatively low intensity, below the lactate threshold, in which PCr breakdown buffers the temporary mismatch between the step increase in ATP demand and the slower (usually exponential) increase in oxidative ATP synthesis, with negligible contribution by glycolytic ATP synthesis; black PCr and pH lines depict behavior during ‘mixed’ exercise, i.e. exercise at higher intensity, above the lactate threshold, in which glycolytic ATP synthesis is also appreciable^11^. The rates of ATP generation by PCr breakdown, glycolysis (*L*) and oxidative phosphorylation (*Q*) must equal ATP demand (*U*), which is shown here as constant for constant-intensity exercise below ‘critical power’^11^: the notional pattern of *L* and *Q* illustrated here shows an intermediate case where glycolytic ATP synthesis is not negligible, but is transient, so that by the end of exercise ATP is supplied only by oxidative ATP synthesis. During recovery from exercise at any intensity, PCr is replenished at the expense purely of oxidative ATP synthesis, the kinetics being exponential at low exercise intensity, following more complicated kinetics after exercise where pH has significantly fallen. The changes in pH in exercise result from the balance between the alkalinizing effect of PCr breakdown (dominant at the start), the progressive acidifying effect of glycolytic ATP synthesis, and the alkalinizing (or acidification-moderating) effect of net acid efflux; during recovery pH is restored by net acid efflux despite the H^+^-generation which accompanies PCr resynthesis (whose early post-exercise dominance can cause a transient initial-recovery acidification)^11^.

**Figure 2:**
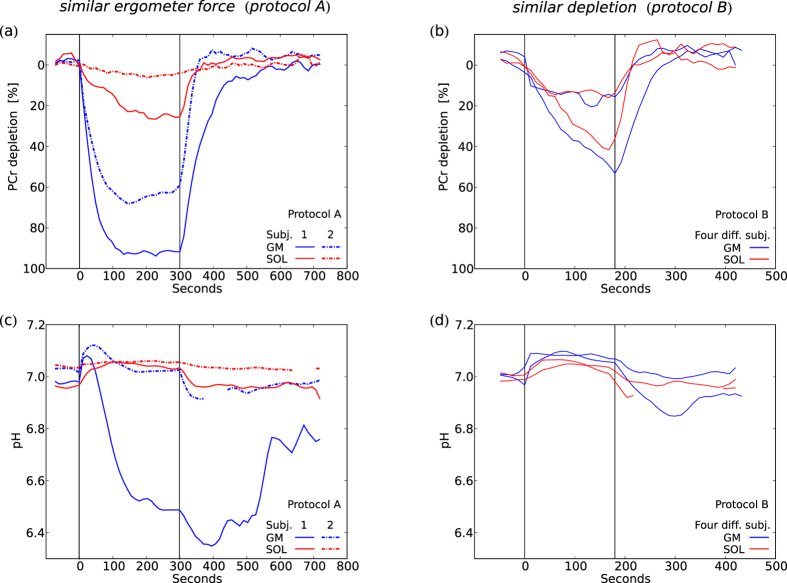
Evolution of PCr (**a,b**) and pH (**c,d**) during exercise and recovery. Data from GM and SOL which were acquired at the same ergometer force, i.e. applying protocol A, are shown for two representative subjects (**a,c**). Time courses representing similar end-exercise PCr depletion in both muscles (two pairs with high or low depletion in each) were selected from data acquired in four different subjects, (**b,d**). This was achieved by applying different ergometer forces, i.e. using protocol B. Vertical lines indicate start and end of exercise.

**Figure 3:**
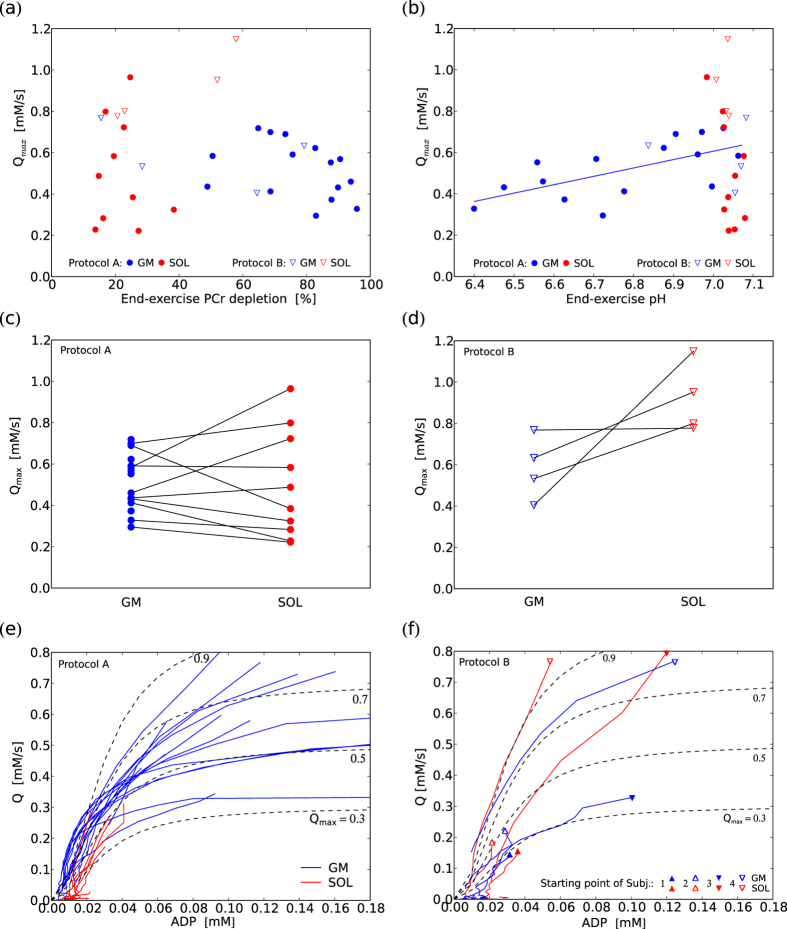
*Q*_max_ of all subjects from protocol A (40% MVC ergometer force) and of four subjects from protocol B (different ergometer forces), selected by having reached similar PCr depletion in both muscles: *Q*_max_ vs. end-exercise values of PCr depletion (**a**) and pH (**b**). *Q*_max_ in GM and SOL in protocol A (**c**) and protocol B (**d**). Evolution of *Q* with [ADP] during recovery in protocol A (**e**) and the selected subjects from protocol B (**f**). Dashed lines depict the theoretical relation according to the ADP-model, at *Q*_max_ of 0.3, 0.5, 0.7 and 0.9 mM/s.

**Figure 4:**
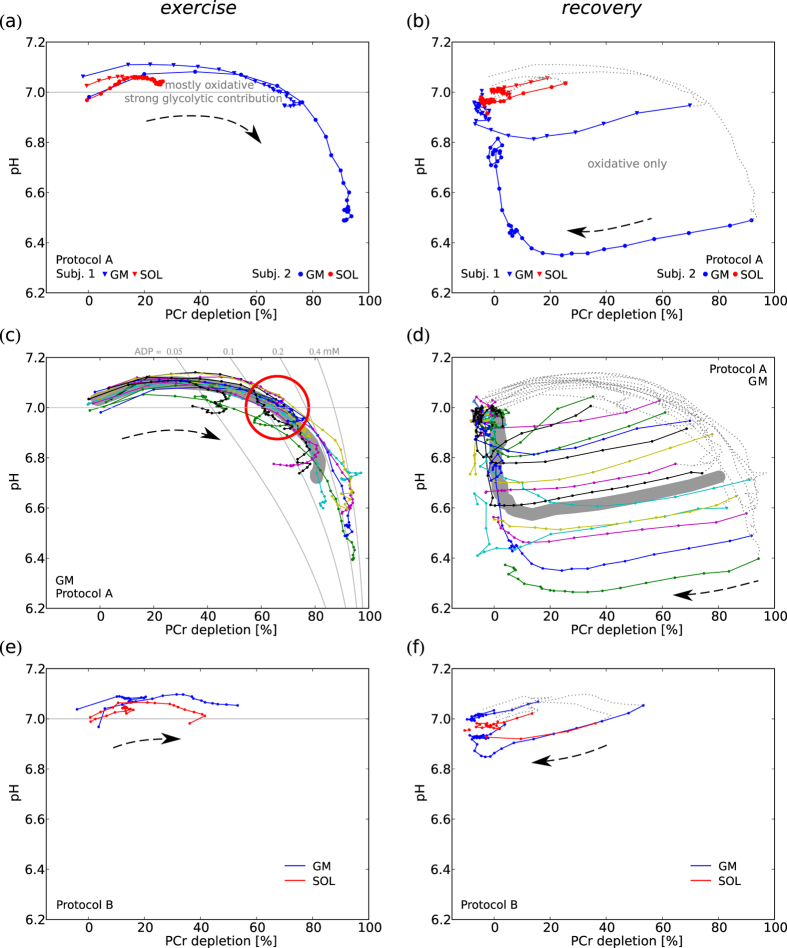
pH vs. PCr depletion during exercise (left) and recovery (right). Data from GM and SOL at the same ergometer force of two representative subjects (**a,b**), and GM of all 15 datasets measured with protocol A (**c,d**). Group averages are depicted as underlying wide dark-grey trajectory (datasets without acidification excluded). Grey solid lines represent neutral pH and ADP isolines, the red circle indicates the PCr depletion range at the starting acidification (in line with the ‘lactate-threshold’ model). Exemplary data from protocol B with ~20% and ~50% end-exercise PCr depletion from each muscle (**e,f**), same datasets as shown in Fig. 2b,d. Arrows indicate direction of temporal evolution, dashed grey lines in recovery plots depict prior evolution during exercise. Spectra of two consecutive time points were averaged, successive data points are equidistant in time (every 12 s).

**Figure 5:**
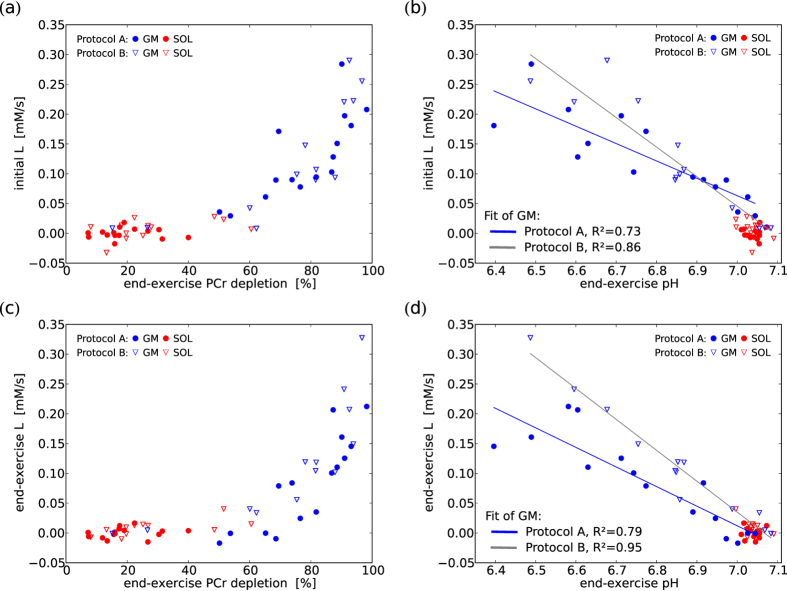
Glycolytic ATP synthesis rate (*L*), as calculated from the time-derivatives of PCr and pH evolution, is very low at exercises resulting in low PCr depletion. Above about 65% a higher end-exercise PCr depletion is correlated with much higher *L*, both for initial (**a**) and end-exercise (**c**) values of *L*, which is in line with the ‘lactate-threshold’ model. A linear regression analysis shows that *L* is correlated linearly with end-exercise pH, again both initial (**b**) and end-exercise *L* (**d**). Initial values of *L* are quantified at 45 s of exercise (cf. grey vertical line in Fig. 6) ensuring absence of *E* and all its associated uncertainties (cf. Eq. 10). Protocol A: 5 min exercise, Protocol B: 3 min exercise. Two spectra averaged per data point before processing.

**Figure 6:**
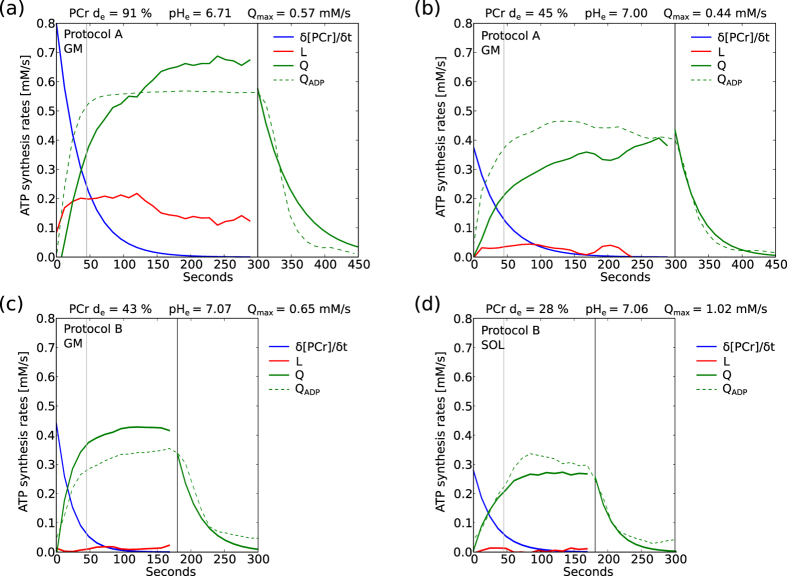
ATP synthesis rates during and after exercise, at strong (**a**) and medium (**b**) activation measured in GM using protocol A, and at low activation measured in GM (**c**) and SOL (**d**) using protocol B. *L* (red) and *Q* (green) were calculated from the derivatives of PCr (blue) and pH time courses, and corrected for H^+^ efflux which was assumed to be linear with pH (Eqs 13 and 14). *Q*_ADP_ (dashed) was quantified using the ADP time courses (Eqs 4 and 5) and is shown for comparison. The black vertical lines mark the end of exercise, the grey lines at 45 s indicate the moment defining initial *L* used in Fig. 5. Two averages per time point (12 s temporal resolution).

**Figure 7:**
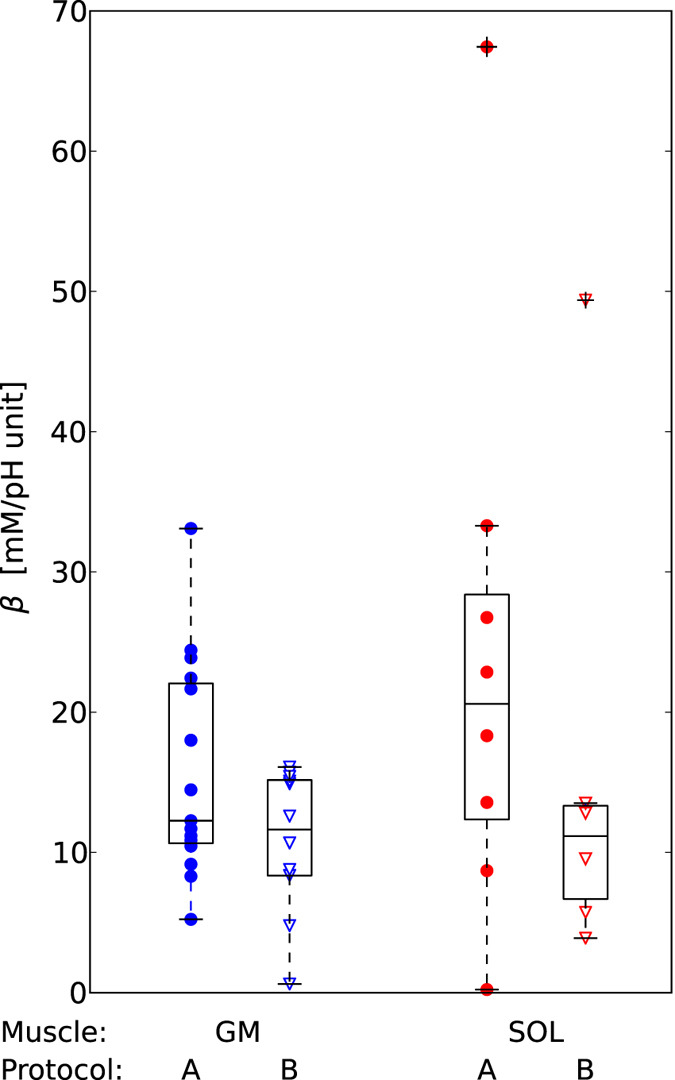
Individual cytosolic buffer capacity *β* was non-invasively quantified from the change in pH and PCr within the first 6 seconds of exercise, where contribution by *L* is considered to be negligible. This was successful in most measurements (see text), and yielded realistic results.

**Figure 8:**
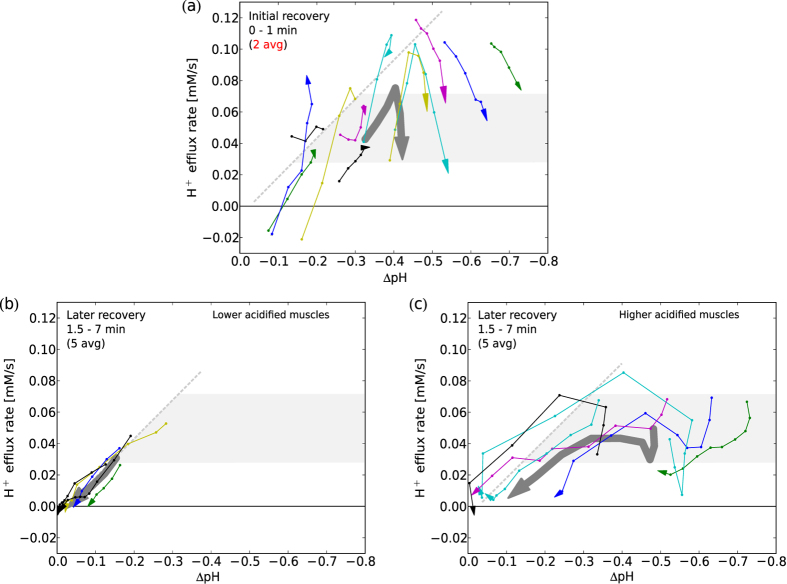
In early recovery (**a**) the individually quantified proton efflux rates *E* showed a strong increase at acidifications below ΔpH ≈ −0.4, whereas at higher acidifications *E* was decreasing (the arrows indicate the direction of temporal evolution). In later recovery (**b,c**), after lowering acidification below about ΔpH ≈ −0.2, the *E* showed approximately linear correlation with acidification (shown for comparative purposes as grey dashed line), supporting the applicability of the linear *E* estimation during exercise (cf. Eq. 13). *E* was calculated from derivatives of pH and PCr evolutions (cf. Eq. 11) using 2x averaged data in initial recovery (**a**) and 5x averaged data in later recovery (**b,c**) where SNR nearly vanishes, especially in measurements at higher acidification (**c**). All data from GM, protocol A (same color-code as in Fig. 4c,d), 3 measurements not included for lack of acidification. Successive data points are equidistant in time with 12 s in (**a**) and 30 s in (**b,c**). The fat grey trajectory depicts *E* calculated from group averages of PCr and pH (**a**).

**Figure 9:**
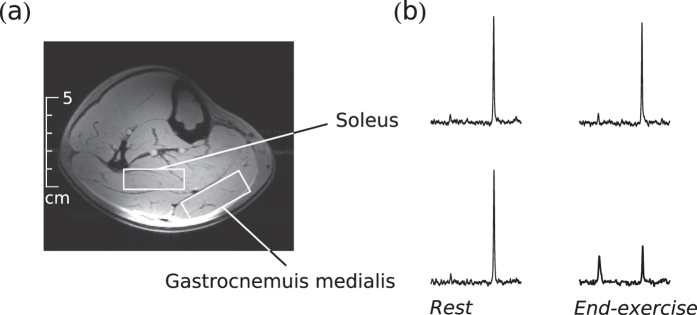
Typical position of the voxels in soleus and gastrocnemius medialis muscle, respectively, for localized ^31^P -MRS at 7 Tesla (**a**). Unaveraged sample spectra of the respective muscles at rest and at the end of exercise (**b**) show the prominent PCr peak and the smaller Pi peak.

To illustrate the time course and the relationship between the quantified parameters, Fig. 1 shows a schematic overview of the expected temporal evolutions of PCr, pH and contributions to ATP synthesis rates. pH and PCr content are quantified by ^31^P-MRS, and their evolution is closely linked to the evolution of ATP synthesis.

During exercise, pH shows an initial increase, caused by the protons consumed by the PCr breakdown, as yet relatively unopposed by the protons arising from glycolytic ATP synthesis. The subsequent fall of pH is driven by the increasingly dominant proton production resulting from glycolytic ATP synthesis (*L*), and mitigated by the proton efflux from the cytosol to the blood (*E*). Total ATP demand (*U*) during exercise has to be covered by the sum of PCr breakdown, *L* and oxidative ATP synthesis (*Q*). During recovery PCr is resynthesized, at the expense of *Q*, and the rate of *δ*[PCr]/*δt* basically reflects *Q*. PCr resynthesis produces protons, which (further) decrease pH at initial recovery, before proton efflux becomes the dominant influence, resulting in the restoration of pH to the basal level.

### Computations

pH values were calculated from the chemical shift, i.e. frequency difference, between Pi and PCr^34^. PCr and Pi signal intensities, representing the area under the peak in the frequency domain, were normalized to basal PCr signal, assuming resting PCr in the cytosol of 33 mmol/L^35^. The PCr recovery rate constant *k*_rec_ and the fraction of depleted phosphocreatine at the end of exercise (i.e. PCr depletion *d*) were determined by fitting a monoexponentional function to the PCr recovery time course^4^,^36^:


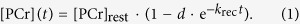


The maximum oxidative capacity of the mitochondria was calculated using an ADP-control model:


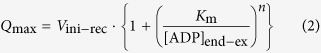


with the initial rate of PCr resynthesis (at *t* = 0 of recovery):


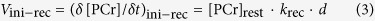


and


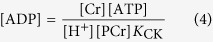


as described in refs 4 and 11, using a Hill coefficient of *n* = 2, and making common assumptions of exchange constants (*K*_m_ = 30 μM, *K*_CK_ = 1.66 × 10^9^ l/mol) and basal concentrations ([PCr]_rest_ = 33 mM, [ATP] = 8.2 mM) contained therein and in ref. 35. This ADP-control model takes into account that PCr resynthesis interacts with pH. Hence, when end-exercise pH is appreciably below basal, the assumptions which underpin the simple linear model of *Q*_max_ are rendered unreliable. The temporal evolution of the oxidative rate of ATP synthesis (*Q*) is calculated using this model as:


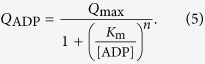


A more direct way of calculating rates of oxidative (*Q*) and glycolytic (*L*) ATP synthesis employs the ATP and H^+^ balance. Total ATP demand (*U*) has to be covered by PCr breakdown, *L* and *Q*:


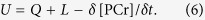


The outcome of H^+^ producing and consuming processes minus the H^+^ efflux from the cell (*E*) has to be buffered by the cytosol:


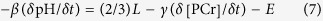


where *β* is the total buffer capacity of the cytosol^11^, and *γ* is a negative stoichiometric coefficient arising from the pH-dependent difference in charge^37^,^38^. Assuming total ATP demand to be constant throughout the (constant-power) exercise, it can be calculated from the first few seconds of exercise, where contributions from *Q* and *L* are still negligible (see Fig. 1). From Eq. 6 this can be read as:


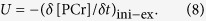


To calculate *L* during exercise Eq. 7 can be rewritten as:


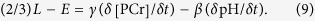


Because the proton efflux is not directly accessible, the calculation of *L* may contain an unknown contribution from *E*. In early or low level exercise there is no acidification in the cytosol. Therefore the proton efflux from the cell to the surrounding blood is assumed to be negligible and *L* can be calculated using Eq. 9 with *E* = 0:


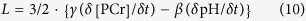


Conversely, during recovery there is assumed to be negligible glycolytic contribution to ATP synthesis, hence in this regime *E* can be directly calculated using Eq. 9 with *L* = 0:


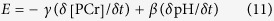


At the very end of exercise the pH value in the cytosol is the same as at the beginning of recovery. Assuming that, consequently, the proton efflux is also the same, the amount of *L* at the end of exercise can be estimated by calculating the value of *E* at initial recovery (*E*_ini-rec_) and applying that to Eq. 9:


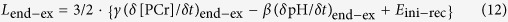


This estimation can be further extended for values during exercise by assuming *E* to be approximately proportional to the acidification given by ΔpH^4^ (note that there is no known influx of protons into the cell, so positive values of ΔpH are discarded for calculation of *E*, e.g. at early exercise or low muscle activation). Obtaining the proportionality factor (*m*_*E*_) from end-exercise values *E*_end-ex_ = *m*_*E*_ΔpH_end-ex_ allows to estimate *L* during exercise using Eq. 9:


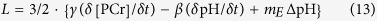


Oxidative ATP synthesis rate (*Q*) during exercise can then be calculated from Eq. 6 using the estimation of *L* in Eq. 13:


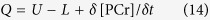


and during recovery, where both *U* and *L* are zero, simply as


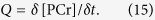


At the very beginning of exercise *L* and *E* are zero, therefore the cytosolic buffer capacity can be calculated using Eq. 7:


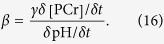


To make sure that the quantification of *β* is free from influence of *L*, only the first data point after the onset of exercise (i.e. after 6 s) was used, in unfiltered, unaveraged data.

If applicable, paired t-tests were used to compare groups, correlations were calculated using linear regression or Spearman’s rank correlation coefficient. Differences and correlations were considered statistically significant for *p* < 0.05.

## Additional Information

**How to cite this article**: Fiedler, G. B. *et al*. Skeletal muscle ATP synthesis and cellular H^+^ handling measured by localized ^31^P-MRS during exercise and recovery. *Sci. Rep.* **6**, 32037; doi: 10.1038/srep32037 (2016).
